# Global transcriptional responses of fission and budding yeast to changes in copper and iron levels: a comparative study

**DOI:** 10.1186/gb-2007-8-5-r73

**Published:** 2007-05-03

**Authors:** Gabriella Rustici, Harm van Bakel, Daniel H Lackner, Frank C Holstege, Cisca Wijmenga, Jürg Bähler, Alvis Brazma

**Affiliations:** 1 EMBL Outstation-Hinxton, European Bioinformatics Institute, Cambridge CB10 1SD, UK; 2 Cancer Research UK Fission Yeast Functional Genomics Group, Wellcome Trust Sanger Institute, Hinxton, Cambridge CB10 1HH, UK; 3 Complex Genetics Group, UMC Utrecht, Department of Biomedical Genetics, 3584 CG Utrecht, The Netherlands; 4 Genomics Laboratory, UMC Utrecht, Department for Physiological Chemistry, 3584 CG Utrecht, The Netherlands; 5 Genetics Department, University Medical Center Groningen, Groningen, The Netherlands

## Abstract

**Background:**

Recent studies in comparative genomics demonstrate that interspecies comparison represents a powerful tool for identifying both conserved and specialized biologic processes across large evolutionary distances. All cells must adjust to environmental fluctuations in metal levels, because levels that are too low or too high can be detrimental. Here we explore the conservation of metal homoeostasis in two distantly related yeasts.

**Results:**

We examined genome-wide gene expression responses to changing copper and iron levels in budding and fission yeast using DNA microarrays. The comparison reveals conservation of only a small core set of genes, defining the copper and iron regulons, with a larger number of additional genes being specific for each species. Novel regulatory targets were identified in *Schizosaccharomyces pombe *for Cuf1p (*pex7 *and SPAC3G6.05) and Fep1p (*srx1*, *sib1*, *sib2*, *rds1*, *isu1*, SPBC27B12.03c, SPAC1F8.02c, and SPBC947.05c). We also present evidence refuting a direct role of Cuf1p in the repression of genes involved in iron uptake. Remarkable differences were detected in responses of the two yeasts to excess copper, probably reflecting evolutionary adaptation to different environments.

**Conclusion:**

The considerable evolutionary distance between budding and fission yeast resulted in substantial diversion in the regulation of copper and iron homeostasis. Despite these differences, the conserved regulation of a core set of genes involved in the uptake of these metals provides valuable clues to key features of metal metabolism.

## Abstract 2

Analysis of genome-wide responses to changing copper and iron levels in budding and fission yeast reveals conservation of only a small core set of genes and remarkable differences in the responses of the two yeasts to excess copper.

## Background

Interspecies comparisons are powerful techniques for gaining insight into biologic processes and their evolution. Accurate annotation of sequenced genomes heavily depends on the availability of gene and protein sequences from other species to allow identification and functional characterization of novel genes by similarity [1,2]. Another area that benefits from interspecies comparisons through the use of cellular and animal model systems is the study of human disease, in which it is often not possible to investigate underlying defects directly. Key to the applicability of these models is the extent to which they accurately reflect the biologic system of interest. Here we address this issue for two metal homeostatic systems, by examining the conservation of transcriptional responses to changing copper and iron levels in budding and fission yeast.

Because of its redox properties, copper is an essential cofactor of many enzymes involved in free radical scavenging, including copper-zinc superoxide dismutase and the respiratory chain (cytochrome *c *oxidase). On the other hand, an excess of free copper can react with oxygen, generating reactive oxygen species that damage cellular components such as nucleic acids, proteins, and lipids. To prevent this from happening, specialized homeostatic mechanisms that tightly control the availability of copper within cells are present in virtually all organisms. These mechanisms have been extensively studied in the budding yeast *Saccharomyces cerevisiae*, and the components involved are highly conserved from prokaryotes to humans [3,4]. The fission yeast *Schizosaccharomyces pombe *provides a complementary model of copper homeostasis. It is estimated that *S. pombe *diverged from *S. cerevisiae *approximately 0.3 to 1.1 billion years ago [5], and many gene sequences are as distantly related between the two yeasts as to their human homologs. A comparison between budding and fission yeast can therefore provide valuable information on the degree to which copper pathways have diverged during evolution.

Copper trafficking in *S. cerevisiae *begins at the plasma membrane, where it is taken up as Cu(I) by the Ctr1p and Ctr3p transporters [6]. Under normal conditions this also requires the action of the ferric/cupric reductases Fre1p and Fre2p [7,8]. Regulation of the copper uptake system is mediated at the transcriptional level by the copper-sensing regulator Mac1p [9-11]. Once in the cytoplasm, copper is shuttled to its target proteins by specific intracellular copper chaperones [12]. One of these chaperones, namely Atx1p, delivers copper to the Ccc2p ATPase in the Golgi system for incorporation into the cuproenzymes Fet3p and Fet5p [13]. These paralogous proteins are multi-copper oxidases that exhibit ferrous oxidase activity and form a high-affinity iron transport complex with the Ftr1p and Fth1p proteins, respectively [14-16]. Copper must therefore be available for the iron transport/mobilization machinery to function, and low copper availability leads to secondary iron starvation in *S. cerevisiae *[17-19].

Similar to copper, iron must be reduced before its uptake at the plasma membrane. This process is partly mediated by the same Fre1p and Fre2p reductases that play a role in copper uptake, together with four additional paralogs (Fre3p to Fre6p) [20-22]. A second, nonreductive iron uptake system involves the four proteins Arn1p to Arn4p, which can acquire iron from siderophore-iron chelates in the medium [23-27]. The intimate link between copper and iron metabolism in *S. cerevisiae *is reflected by the fact that Rcs1p (Aft1p), which is the transcription factor responsible for induction of the iron uptake systems, also regulates *FRE1*, *CCC2*, *ATX1*, *FET3 *and *FET5*, which are involved in copper trafficking [28,29]. A second iron-responsive transcription factor, Aft2p, regulates a subset of Aft1p targets [30], but its role in iron homeostasis is less well understood.

When copper levels are high, *S. cerevisiae *specifically induces expression of *SOD1 *and the *CUP1a/b *and *CRS5 *metallothioneins [31-33]. Metallothioneins represent a group of intracellular, low-molecular-weight, cysteine-rich proteins that sequester free metal ions, preventing their toxic accumulation in the cell. The response to high copper is mediated by the transcriptional regulator Ace1p (Cup2p) [34,35].

Compared with *S. cerevisiae*, copper metabolism in *S. pombe *is less well understood, although homologs to several budding yeast core components have now been experimentally characterized. Three genes encode the high affinity copper uptake transporters: *ctr4 *and *ctr5*, whose products are localized to the plasma membrane, and *ctr6*, which encodes a vacuolar membrane transporter [36]. Expression of these transporters is regulated by Cuf1p, which is functionally similar to *S. cerevisiae *Mac1p [37,38]. Both the reductive and nonreductive iron uptake systems are also present in *S. pombe*. The reductive system consists of the ferric reductase Frp1p, the Fio1p multi-copper oxidase, and the Fip1p permease [39,40], whereas the siderophore-iron transporters are encoded by *str1*, *str2*, and *str3 *[41]. When sufficient iron is available, expression of the reductive and nonreductive uptake systems is repressed by the Fep1p transcription factor [41,42]. Interestingly, in contrast to *S. cerevisiae *Mac1p, the copper-dependent regulator Cuf1p was reported to repress directly the reductive iron uptake system during copper starvation in *S. pombe *[43].

Only two genes have thus far been implicated in resistance to high copper stress in *S. pombe*. These encode the superoxide dismutase copper chaperone Ccs1p [44] and a phytochelatin synthase (PCS) [45]. Phytochelatins are a class of peptides that play an important role in heavy metal detoxification in plants and fungi, but which are absent in *S. cerevisiae*. They are nontranslationally synthesized by PCS from glutathione and can sequester unbound heavy metals. Loss of function of either of the genes encoding Ccs1p or PCS results in increased sensitivity to high copper levels in fission yeast [44,45]. One metallothionein gene, *zym1*, has also been identified in *S. pombe*, but exposure to high copper did not affect its expression level [46]. No transcription factors that regulate the response to high copper have thus far been described.

Global gene expression studies have been insightful in exploring transcriptional responses to stress in both budding and fission yeast [47,48]. To identify novel fission yeast genes that may play a role in copper and iron homeostasis, we used DNA microarrays to evaluate differential gene expression in *S. pombe *cells growing under varying copper and iron levels. The results were compared with data gathered from a similar set of experiments conducted in *S. cerevisiae *[19] in order to determine the extent to which responses to changes in environmental copper levels have diverged between the two yeasts. We show that despite conservation of core elements, significant differences exist in the regulation of copper and iron metabolism genes in budding and fission yeast, in particular in their responses to copper toxicity. Our findings also provide new insights into the coregulation of copper and iron metabolism in *S. pombe*.

## Results

We monitored global gene expression in *S. pombe *wild-type cells in response to changes in environmental copper levels. Two conditions were initially investigated: copper starvation (100 μmol/l bathocuproinedisulfonic acid [BCS], a copper chelator) and copper excess (2 or 25 μmol/l CuSO_4_). These conditions allowed induction of known copper-dependent genes without adverse effects on growth rate that could confound the results. The conditions for copper starvation were chosen based on data from the literature [36,44]. For copper excess, we tested a number of concentrations close to the levels that were known to affect growth in *S. cerevisiae *[19], and selected those that did not negatively affect *S. pombe *growth rate (data not shown). RNA samples were collected at regular intervals after addition of either BCS or CuSO_4 _and compared with untreated wild-type cells by DNA microarray analysis.

### Copper deprivation does not cause significant iron starvation in fission yeast

The classes of genes whose expression was either induced or repressed under copper starvation in fission yeast are listed in Table 1 (also see Additional data file 1 [Supplementary table 1]). A major group of genes upregulated by BCS addition was involved in metal ion uptake, including genes encoding copper transporters, namely *ctr5 *and *ctr6*, which have previously been reported to be induced in states of low copper [36,43,49]. Ctr5p is known to form a functional complex with Ctr4p [49]. The gene for the latter protein was not represented on the arrays, but it was found to be highly induced (>24×) in a real-time quantitative polymerase chain reaction (qPCR) performed on the same samples used for the microarray experiment (Additional data file 1 [Supplementary table 1]).

**Table 1:** Gene classes induced and repressed upon changes in *S. pombe *copper or iron status

Condition	Induced		Repressed	
	
	Classification	Gene number	Classification	Gene number
Low copper (100 mmol/l BCS)	Metal ion transport	5	Oxidoreductases and dehydrogenases	3
	Peroxisomal proteins	2	Flavoproteins	2
	Other transport	1	Antioxidants	2
	Others	3		
Low iron (300 mmol/l FZ)	Metal ion transport	8	Localized to the mitochondrion	6
	Other transport	2	Transporters	3
	Peptide biosynthesis	2	Metal metabolism	2
	Iron-Sulfur cluster assembly	1	Iron/sulfur cluster proteins	2
	Others/Unknown	19	Thiamine biosynthesis	2
			Others/unknown	9
High copper (2 mmol/l CuSO_4_)	Protein folding/chaperone	12	Transporters	7
	Antioxidants	6	Amino acid metabolism and transport	4
	Sulphur amino acid biosynthesis	6	Ribosomal proteins	2
	Carbohydrate metabolism	4	Others/unknown	11
	Stress response	4		
	Iron uptake	3		
	Signaling and transcription regulation	2		
	Lipid biosynthesis	2		
	Peptide biosynthesis	2		
	Other/unknown	28		

BCS, bathocuproinedisulfonic acid; FZ, ferrozine.

A number of predicted flavoproteins, oxidoreductases, and dehydrogenases were downregulated during copper starvation (Table 1). These enzymes catalyze a wide range of biochemical reactions, and their repression may reflect a need for copper in some of these processes. Reduced expression of the antioxidant genes *gst2 *and *sod1*, which encode a glutathione S-transferase and a copper-zinc superoxide dismutase, respectively, is not surprising, considering the aforementioned link between copper and the generation of free radicals. Downregulation of *sod1 *may also result from the reduced availability of copper, which is needed to convert apo-Sod1p to its active form.

**Figure 1:**
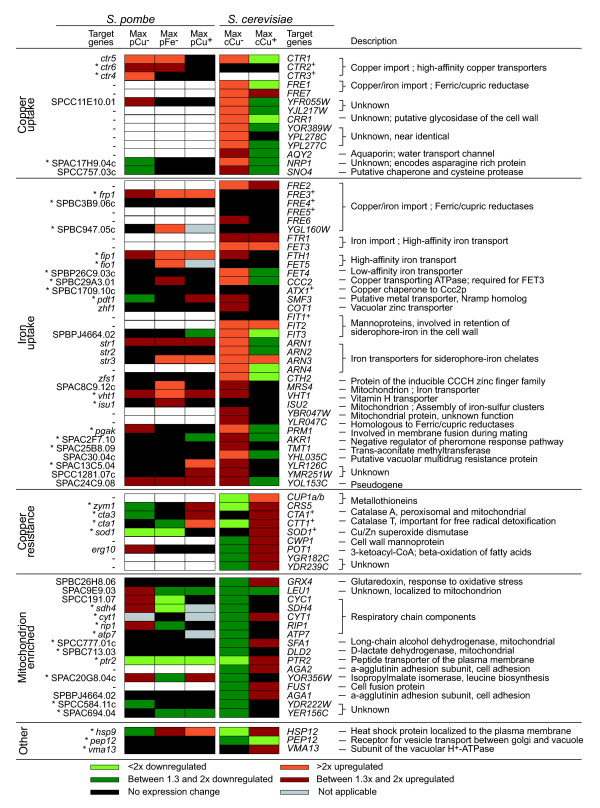
Comparison of copper and iron metabolism between budding and fission yeast. The transcriptional responses of four clusters of *S. cerevisiae *genes identified by Van Bakel and coworkers [19] to changing copper levels are shown in comparison with expression changes in *S. pombe *homologs under similar conditions. Fission yeast genes with curated orthologs in budding yeast are indicated by asterisks. The clusters were supplemented with 10 additional genes that are known to be involved in *S. cerevisiae *copper and iron metabolism (+), as well as three genes found outside these clusters (other) [19]. The maximal fold change in expression over time, as determined from averaged replicates at each time point, is displayed for each gene for the experimental conditions used (*pCu*^-^, low copper, 100 μmol/l bathocuproinedisulfonic acid [BCS]; *pFe*^-^, low iron, 100 μmol/l ferrozine; *pCu*^+^, high copper, 2 μmol/l CuSO_4_; *cCu*^-^, low copper, 100 μmol/l BCS; *cCu*^+^, high copper, 8 μmol/l CuSO_4_). The graded color scale at the bottom indicates the magnitude of expression changes.

**Figure 2:**
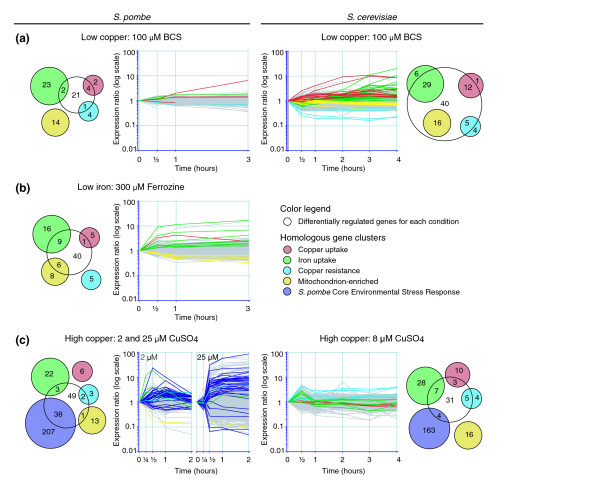
Differences in transcriptional profiles of known copper and iron regulated genes between *S. pombe *and *S. cerevisiae*. The *S. pombe *genes implicated in copper or iron metabolism by homology with *S. cerevisiae *(Table 1) were compared with the set of genes that exhibited expression changes in response to changes in copper or iron levels. Overlaps between these lists indicate conserved regulation and are visualized in Venn diagrams. The central circle in each Venn diagram indicates the total number of differentially expressed genes in conditions of **(a) **low copper, **(b) **low iron, or **(c) **high copper. Individual gene clusters with a role in copper or iron metabolism are shown in different colors. The behavior of homologous genes in *S. cerevisiae *is shown in comparison. The temporal transcriptional profiles for overlapping segments in the Venn diagrams, representing conserved copper and iron dependent gene regulation, are visualized in graphs that plot the averaged expression ratio as a function of time.

Previous expression studies in budding yeast have identified a number of genes that are consistently differentially expressed in varying copper levels [17-19]. For our comparison with fission yeast, we used a recent microarray time-course dataset that closely matches ours with respect to experimental setup, allowing direct comparison between the two yeasts [19]. In this study, four gene clusters were described whose mRNA expression was altered in copper starvation or excess. Three of these clusters contain genes that are involved in copper uptake, copper detoxification, or iron uptake, which are respectively regulated by Mac1p, Ace1p, and Rcs1p/Aft2p (Figure 1). The late induction of the iron regulon in conditions of low copper is thought to result from a secondary iron starvation [17-19]. A fourth cluster was downregulated after prolonged copper deprivation and contains genes that function in the mitochondrion, including a large component of the respiratory chain. Regulation of this latter group is believed to be linked to a dependency on copper or iron by these metabolic processes [19]. Many of the genes that are implicated in copper and iron metabolism in *S. cerevisiae *have homologs in *S. pombe*. For this study we used orthologs from a manually curated list [47]; when these were unavailable, homologs were identified on the basis of sequence similarity. To determine the extent to which the *S. pombe *homologs are similarly controlled at the transcriptional level as their *S. cerevisiae *counterparts, we compared their expression patterns during varying copper conditions. Figure 1 shows a direct comparison between homologous gene pairs in four transcriptional clusters with a specific role in copper or iron metabolism in either yeast. The same gene clusters are used in Figure 2 to summarize how many genes from each group exhibit conserved regulation between *S. pombe *and *S. cerevisiae *in response to changing copper and iron availability. In addition, the expression patterns for homologs that exhibit conserved expression in both *S. pombe *and *S. cerevisiae *are indicated for direct comparison of the timing and amplitude of expression changes.

When evaluating the transcriptional profiles of budding and fission yeast in response to copper deprivation, a striking difference was observed in the number of differentially expressed genes (Figure 2a). Of the four copper responsive gene clusters described in *S. cerevisiae*, major expression changes in *S. pombe *were only observed for homologs to the cluster involved in copper uptake (*ctr4*, *ctr5*, *ctr6*, and SPCC11E10.01; Figures 1 and 2a). The timing of induction of the copper uptake systems is similar in both yeasts, with strong induction of *ctr5 *in *S. pombe *and *CTR1 *in *S. cerevisiae *over a period of 3 hours (Figure 2a). The marked upregulation of the complete iron regulon in *S. cerevisiae*, starting after 2 hours of copper deprivation and peaking at 3 hours, is virtually absent in *S. pombe*, with the exception of *str1 *and *frp1 *(Figures 1 and 2a) [40,41]. Induction of *str1 *was confirmed in three independent microarray experiments, whereas induction of *frp1 *was validated by real-time PCR (data not shown), because of missing data in two experiments. The lack of substantial induction of genes involved in iron uptake suggests that, in the experimental conditions used here, copper deprivation does not lead to a significant secondary iron starvation.

### A core set of iron regulated genes is conserved between the *S. cerevisiae *and *S. pombe*

To identify putative novel genes involved in iron metabolism, we treated *S. pombe *cells with the specific iron chelator ferrozine (300 μmol/l). Iron deprivation caused changes in the expression of 56 genes (Additional data file 1 [Supplementary table 2]), which were of much greater amplitude than was found during copper starvation (Figure 2a,b). Many of the induced genes can be directly linked to iron uptake (eight genes) and processing (one gene), whereas those downregulated are involved in metabolic processes, which is consistent with previous reports on *S. cerevisiae *(Table 1) [19,50]. A large overlap was observed between the cluster of mitochondrial genes in *S. cerevisiae *and their homologs in *S. pombe*, supporting the initial assumption that the changes in this cluster after copper deprivation in budding yeast are linked to secondary iron starvation [19] (Figure 2).

A core set of nine *S. pombe *homologs exhibited conserved regulation as compared with the iron regulon in *S. cerevisiae *(Figure 2b). These include the five previously identified iron regulated genes (*frp1*, *str3*, *fio1*, *fip1*, and *str1*) as well as two predicted novel ones: SPBC947.05c and *isu1*. Both of these can be directly linked to iron metabolism. *Isu1 *encodes a scaffold protein that is involved in mitochondrial iron-sulfur cluster biosynthesis [51]. SPBC947.05c is predicted to encode a ferric reductase similar to Frp1p, suggesting a role in the reduction of iron before its uptake by the Fip1p-Fio1p complex. Two additional genes encoding a vitamin H transporter (*vht1*) and a predicted mitochondrial iron transporter (SPAC8C9.12c) are homologous to genes induced as part of the *S. cerevisiae *iron regulon [17,19,52], but they lack a consensus Fep1p binding site. Considering the conserved regulation between the two yeasts in response to iron deprivation, these genes still represent good candidates for a role in iron metabolism.

An interesting finding was the relatively strong upregulation of *ctr5 *(4.3-fold) together with the iron uptake system, which may occur to ensure the availability of copper for incorporation into the Fio1p oxidase. In the absence of a putative Fep1p binding site in the promoter region, the mechanism behind this induction is as yet unclear.

### Identification of novel regulatory targets for Cuf1p and Fep1p

**Figure 3:**
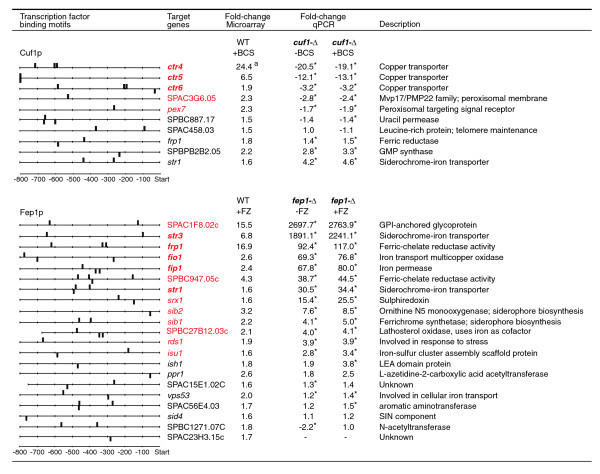
Novel target genes for Fep1p and Cuf1p. The expression of genes induced during copper and iron starvation and containing one or more putative Cuf1p and Fep1p binding motifs in an 800 base pair promotor region was evaluated by real-time quantitative polymerase chain reaction (qPCR) in strains deleted for either Cuf1p or Fep1p. The fold change in target gene expression in *fep1*-Δ and *cuf1*-Δ mutants is shown relative to a wild-type control. The deletion strains were grown in yeast extract (YE) medium, with or without copper or iron chelator added as indicated (± BCS, with or without addition of 100 μmol/l bathocuproinedisulphonate; ± FZ, with or without addition of 300 μmol/l ferrozine). Wild-type control strains were grown in YE medium without metal chelator. Averaged fold changes were obtained by qPCR for two biologic replicates, assayed in duplicate. Significant expression changes (*P *≤ 0.05) determined in a two-sided Student's *t *test are indicated by asterisks. High confidence transcription factor target genes are indicated in red; previously known targets are shown in bold. The maximum observed fold change during the microarray time course, as determined from averaged replicates, is shown in comparison. ^a^Value obtained by quantitative real-time PCR.

The genes induced during copper and iron starvation represent putative novel target genes for the transcription factors Cuf1p and Fep1p, respectively. However, these expression changes can also be the result of additional regulatory mechanisms, given the involvement of copper and iron in several metabolic pathways [53]. We therefore searched for Cuf1p and Fep1p binding motifs upstream of 11 genes that were upregulated in low-copper conditions and 32 genes that were upregulated in low-iron conditions (Additional data file 1 [Supplementary tables 1A and 2A]). Seven genes contained one or more copies of the CuSE binding motif, which may reflect direct regulation by Cuf1p (Figure 3). Putative Fep1p binding motifs were found in 21 genes, including five out of the six genes encoding previously identified Fep1p targets (*fip1*, *frp1*, *fio1*, *str1*, and *str3*; Figure 3) [41]. Most of these genes contain multiple putative Fep1p binding sites, although it has been shown that only one of these motifs is sufficient to confer iron dependent regulation by Fep1p [42].

The role of Fep1p and Cuf1p in regulating the putative novel target genes was further evaluated by examination of the expression levels of these genes in *cuf1*-Δ and *fep1*-Δ mutants using qPCR (Figure 3). For this purpose, the deletion strains and a wild-type control were grown in yeast extract (YE) rather than Edinburgh minimal medium (EMM) medium, because *cuf1*-Δ and *fep1*-Δ growth was found to be impaired in the latter medium [42]. Genes were considered valid Cuf1p targets when they exhibited a significant (*P *≤ 0.05) and greater than 1.5-fold decrease in expression relative to the wild-type control. The same cut-offs were used to identify putative Fep1p targets, with the exception that induced genes were considered instead, which is consistent with the role of Fep1p as a repressor. We further subjected the *cuf1*-Δ and *fep1*-Δ mutants to conditions of copper and iron deprivation, respectively. The absence of significant additional expression changes relative to standard conditions (Figure 3) confirms that the observed target gene regulation is indeed conferred by the copper or iron responsive transcription factors, as opposed to indirect effects related to a reduction in metal availability.

Based on our stringency cut-offs, we can identify two novel Cuf1p targets, namely *pex7 *and SPAC3G6.05, both of which are predicted to encode peroxisomal proteins. This strongly suggests a role for this organelle in *S. pombe *copper homeostasis, perhaps linked to its function in reactive oxygen species metabolism [54]. Consistent with previous observations [43], *frp1 *and *str1 *were significantly induced in the *cuf1*-Δ mutants. This probably results from a secondary iron starvation in *S. pombe *and is further discussed below.

The eight novel regulatory targets for Fep1p exhibit a clear functional link to iron metabolism. The genes *sib1 *and *sib2 *both encode proteins that were previously implicated in siderophore biosynthesis [55], and our findings confirm that *S. pombe *induces production of siderophores in iron limiting conditions. The expression of *isu1 *points to a link to iron-sulfur biosynthesis, which may further involve the sulfiredoxin Srx1p. SPBC947.05c is predicted to have ferric-chelate reductase activity based on sequence similarity, and it is expected to play a role in iron reduction before uptake, analogous to Frp1p. The role of the remaining proteins (Rds1p, SPAC1F8.02c, and SPBC27B12.03c) in iron homeostasis is currently unclear. The considerable induction of SPAC1F8.02c, greater than that for all previously identified Fep1p targets, indicates that this glycoprotein plays an important role in iron uptake.

### *S. pombe *responds to high copper levels with a general stress response

Exposure of fission yeast to limited copper stress (2 μmol/l CuSO_4_) resulted in a rapid (within 15-30 min) but transient transcriptional response involving 93 genes (Figure 2c and Additional data file 1 [Supplementary table 3]). When copper levels were increased to 25 μmol/l CuSO_4_, this number rose dramatically to 1,259 genes, and the expression changes persisted for the 2-hour time course, reaching a plateau after 30 min (Figure 2c). The size of the response suggests additional cell stress at these copper levels and is likely to result from secondary effects of elevated copper levels. Considering that *S. pombe *is able to sustain growth in copper concentrations up to 10 mmol/l [56] and that growth rate was not impaired compared with standard conditions (data not shown), the observed expression changes indicate a physiologic response to copper rather than cytotoxic effects. We focused on the genes that were also differentially expressed in the limited copper experiment, because they were the first to respond to high-copper stress and are therefore more likely to represent direct copper-specific regulation.

The global character of the *S. pombe *gene expression response to medium and high copper levels is in stark contrast to the limited expression changes found in *S. cerevisiae *cells treated with copper (Figure 2c). Notably, the changes in fission yeast already occur at much lower levels of copper (2 μmol/l versus 8 μmol/l). The genes that are induced by high copper levels are involved in a variety of functions (Table 1). As expected, these include antioxidants with an established role in heavy metal detoxification such as glutathione *S*-transferase (SPAC688.04c and SPCC965.07c), thioredoxin (SPBC12D12.07c and *trx2*), zinc metallothionein (*zym1*), and superoxide dismutase (*sod1*).

Interestingly, a number of iron uptake genes, including *frp1*, *str1*, and *fip1*, were induced in response to high copper (Figures 1 and 2c), which is consistent with previous findings [43]. A small and transient induction of iron metabolism genes was also observed in budding yeast, peaking after a 30 min exposure to 8 μmol/l CuSO_4 _(Figure 2c). The same group, however, is also known to be upregulated in response to other stressors such as cadmium or hydrogen peroxide, with the exception of *fip1*, which is downregulated [47]. Regulation of these genes may therefore be the result of general stress and unrelated to copper metabolism. Another possible explanation for the induction of iron regulon genes is that excess copper triggers iron starvation by competing with iron uptake. It is known that the low-affinity Fet4p iron transporter in *S. cerevisiae *can be inhibited by elevated concentrations of cobalt and cadmium [57]. Fet4p and its *S. pombe *ortholog (SPBP26C9.03c) may well be similarly affected by copper.

A large proportion of the genes (41%) exhibiting changes in high copper are part of the core environmental stress response (CESR) [47], which is known to be activated in response to several distinct stress conditions (Figure 2c). The major conserved regulators of this general stress response in *S. pombe *that have been identified to date are the Sty1p kinase and the transcription factor Atf1p. Sty1p is turned on as part of a mitogen-activated protein kinase cascade by a variety of stressors [58-62]. The resulting transcriptional changes are effected, at least in part, by Atf1p, which is phosporylated by Sty1p [63-67]. The majority of the induced CESR genes were indeed part of the set of known Sty1p or Atf1p regulated genes (27 out of 38) [47], suggesting an important role for these proteins in the regulation of at least part of the response to high copper.

Considerable overlap was also found with genes previously described to be induced in response to the heavy metal cadmium [47], and almost all of the genes expressed in response to high copper were also induced by cadmium (data not shown). In particular, genes involved in the sulfur amino acid biosynthetic pathway (Table 1 and Additional data file 1 [Supplementary table 3A]), which is required for both glutathione and phytochelatin synthesis, were upregulated in both experiments. Expression of the *S. pombe *phytochelatin synthase itself (SPAC3H1.10) could not be determined because it did not produce measurable signals at most time points.

Our results further underscore the general nature of the *S. pombe *response to high copper, even when only a relatively small subset of genes that reacted early to copper stress is considered. From comparisons with previous microarray experiments in *S. pombe *subjected to environmental stresses [47], however, we can identify a small subset of genes that are specifically downregulated in response to high copper (*ptr2*, SPBC13A2.04c, SPAP7G5.06, SPAC5H10.01, SPCC132.04c, SPCC1223.09, SPAC11D3.18c, SPAC11D3.15, and SPAC1039.08). Most of these genes are involved in amino acid metabolism.

### *S. cerevisiae *cannot compensate for the loss of Ace1p with a general stress response

Wild-type *S. cerevisiae *is protected from copper stress by the presence of metallothioneins; when copper concentration increases, induction of metallothionein synthesis is sufficient to neutralize the toxic effect of the metal and prevent oxidative stress. This can be inferred from absence of additional stress induced genes in the *S. cerevisiae *response to high copper levels [19] (Figure 2c). When metallothionein synthesis cannot be initiated (for example, because of lack of the transcription factor responsible for their activation, as in an *ace1*-Δ strain), free copper can exert its toxic effect on cellular components, leading to reduced tolerance to high copper [68]. Because *S. pombe *responds to metal accumulation by initiating a general stress response, we were interested to determining whether *S. cerevisiae *has retained the ability to induce a similar response in the absence of the specific high-copper detoxification system.

**Figure 4:**
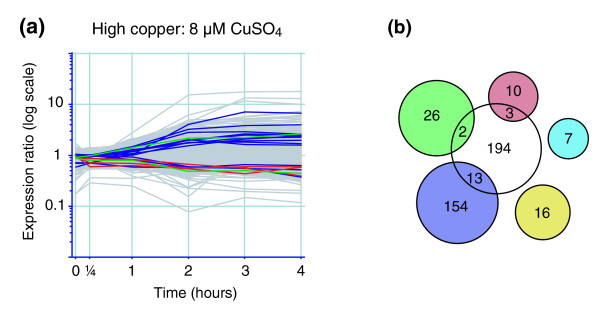
*S. cerevisiae ace1*-Δ mutants fail to induce a core environmental stress response in response to high copper. **(a) **Transcriptional response of *S. cerevisiae ace1*-Δ mutants to excess copper (8 μmol/l CuSO_4_). **(b) **Venn diagrams showing the overlap between differentially expressed genes in the *ace1*-Δ mutants (Figure 3a), and clusters of genes that are orthologs to the core environmental stress response in fission yeast, or known to be regulated in response to copper or iron. Venn diagrams and transcriptional profiles are colored as in Figure 2.

Although deletion of *ACE1 *resulted in a drastic increase in the number of genes that respond to copper stress (212 versus 50 in wild-type cells) as well as the magnitude of their changes (Additional data file 1 [Supplementary table 4]), there were significant differences in the types of genes regulated (Figure 4). Only 6% of the differentially expressed genes were orthologous to the CESR group (named ESR/CER in *S. cerevisiae*), which accounts for 41% of the *S. pombe *response to high copper. Even when considering all genes of the *S. cerevisiae *ESR/CER [48,69], this number increases only slightly to 8%. We also directly compared the fission yeast genes induced by high copper levels in the wild-type with those induced in budding yeast *ace1*-Δ, and we found that only 18 orthologous genes were differentially expressed in both experiments.

Two major classes of genes were induced upon copper stress in *ace1*-Δ mutants, encoding components of the proteasome and stress response proteins (Table 2). Similar induction of proteasome related genes have been observed in response to diamide (a sulfhydryl oxidizing agent), griseofulvin (antifungal agent), and methyl methanesulfonate (a DNA damaging agent) [48,70,71] and may be indicative of severe stress leading to cell death. The reduction in growth rate observed for *ace1*-Δ mutants during the 4 hours of exposure to 8 μmol/l CuSO_4 _is consistent with this hypothesis. Expression of proteasome genes is also highly induced in *S. pombe *cells exposed to 25 μmol/l CuSO_4 _(data not shown). Taken together, our findings indicate that *S. cerevisiae ace1*-Δ mutants exhibit a different response to high copper as compared with *S. pombe*, and this discrepancy may be an important contributing factor to the copper hypersensitivity that has been observed in these mutants [68]. Thus, *S. cerevisiae *cells can only poorly compensate for the absence of metallothioneins, whereas *S. pombe *cells may have adapted to the lack of a *CUP1 *ortholog by launching a general stress response.

**Table 2:** Gene classes induced or repressed by 8 μmol/l CuSO_4 _in *S. cerevisiae cup2*-Δ mutants

Induced		Repressed	
Classification	Gene number	Classification	Gene number

Protein catabolism/proteasome	38	Transport	13
Response to stress	26	Amino acid and derivative metabolism	10
Transport	12	Carbohydrate metabolism	5
Organelle organization and biogenesis	4	Response to stress	3
Protein modification	6	Lipid metabolism	3
Protein biosynthesis	5	Transcription	2
Transcription	3	Others/unknown	39
Others/unknown	43		

### *S. cerevisiae *metallothionein improves *S. pombe *copper tolerance

**Figure 5:**
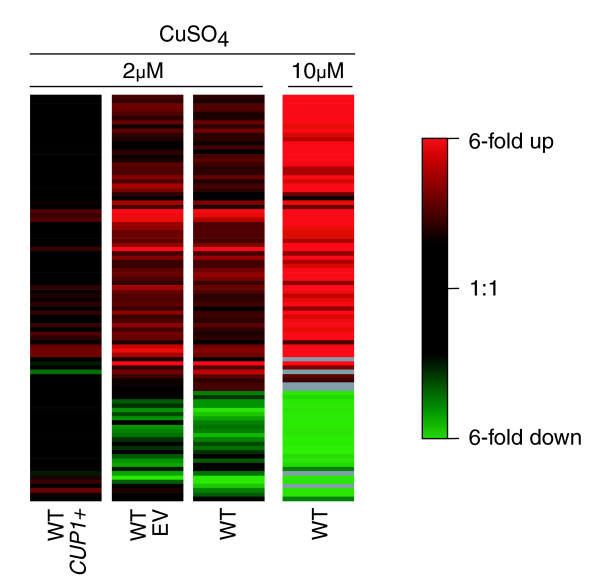
Expression of Cup1p in *S. pombe *reduces the effects of high copper stress. Diagram of expression patterns in fission yeast overexpressing *S. cerevisiae CUP1 *or an empty control vector (EV) after exposure to 2 μmol/l CuSO_4 _for 30 min. The profiles for wild-type (WT) fission yeast in response to 2 and 10 μmol/l CuSO_4 _are shown for comparison. Data are displayed for the set of 93 genes that were differentially expressed in the 2 μmol/l CuSO_4 _experiment after hierarchical clustering.

To test the possibility that expression of an exogenous metallothionein gene could reduce the fission yeast stress response after exposure to high copper levels, the budding yeast *CUP1 *gene was over-expressed in fission yeast. Intriguingly, genes induced in wild-type *S. pombe *cells in response to high copper levels were less induced in a strain over-expressing *CUP1 *(*leu1-32 h*^- ^pREP3X-CUP1). Similar levels of induction were detected between the wild-type and the control strain over-expressing the vector only (*leu1-32 h*^- ^pREP3X; Figure 5). Consistent with these findings, *CUP1 *over-expressing cells (but not cells over-expressing the vector only) were able to grow on EMM plates containing 0.1 mmol/l CuSO_4 _(data not shown). We conclude that the budding yeast *CUP1 *gene greatly helps fission yeast to cope with excess copper.

## Discussion

The work presented in this report provides an overview of transcriptional programs of fission yeast in response to changing copper and iron levels. We identify two novel candidate genes regulated by Cuf1p and a further eight regulated by Fep1p; additional putative regulatory targets were detected with lower confidence. Our results support the view that *S. pombe *reacts to a variety of different stresses by activating a core set of CESR genes. Substantial overlap was found between copper and cadmium stress [47], suggesting that both metals have similar effects on *S. pombe *gene expression, which may be triggered by the resulting oxidative stress rather than by direct metal sensing.

The comparison between budding and fission yeast reveals conservation of relatively small, core copper and iron regulons, with a larger number of additional genes that are specific to each yeast. Of the 13 copper or iron responsive *S. pombe *genes with homologs in the *S. cerevisiae *copper and iron regulons, 10 encode proteins that are directly involved in metal uptake and trafficking (*ctr4*, *ctr5*, *ctr6*, *fip1*, *fio1*, *frp1*, *str1*, *str3*, SPBC947.05c, and SPAC8C9.12c). The function of the other three genes (SPCC11E10.01, *vht1*, and *isu1*) is less well understood, but their conserved regulation suggests an important role in metal metabolism. SPCC11E10.01 is the fission yeast counterpart to YFR055W, which encodes a protein of unknown function and has been reported as a Mac1p target in a number of microarray studies in budding yeast [17-19]. The mitochondrial iron-sulfur cluster assembly protein *isu1 *and its *ISU2 *ortholog are of particular interest, because iron-sulfur cluster synthesis in the mitochondrion has been linked to iron sensing by the Rcs1p transcription factor in *S. cerevisiae *[72]. It is therefore tempting to speculate that these genes have a conserved regulatory role for the iron regulons of *S. pombe *and *S. cerevisiae*.

The genes that are uniquely regulated in each yeast in response to iron or copper starvation mainly encode proteins that are involved in metabolic processes that depend on these metals. When considering only validated Cuf1p and Fep1p target genes, we found possible involvement of the peroxisome in *S. pombe *copper homeostasis that has not been observed in *S. cerevisiae*. Unlike budding yeast, fission yeast has also retained the ability to induce siderophore biosynthesis genes. Finally, the presence of a number of *S. pombe *iron regulon genes with an as yet unknown role in iron homeostasis reflects the evolutionary divergence between the two yeasts.

Several homologs to genes that are highly induced during budding yeast iron starvation were not differentially expressed when fission yeast was subjected to similar conditions. This includes the *S. pombe *homolog (SPBPJ4664.02) to the *S. cerevisiae FIT1*, *FIT2*, and *FIT3 *genes, which encode proteins that are believed to trap iron in the cell wall. It is therefore unlikely that a similar mechanism exists in fission yeast. The fission yeast ortholog to the *S. cerevisiae *Ccc2p ATPase was not picked up as differentially expressed in iron deprived conditions, but only failed to reach our threshold by a narrow margin.

**Figure 6:**
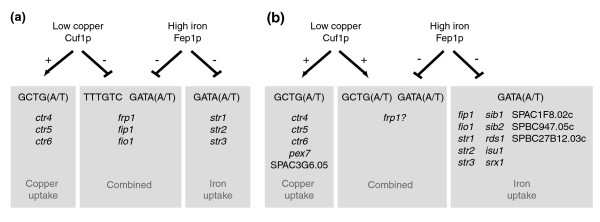
An updated model for transcriptional regulation by Cuf1p and Fep1p. **(a) **Previously proposed mechanism for Cuf1p-dependent repression of iron uptake genes [43]. **(b) **Revised model of Cuf1p and Fep1p regulation in *S. pombe*, including novel regulatory targets. Details are given in the text.

In response to copper deprivation, we found that *frp1 *and *str1 *are upregulated, suggesting a positive link between copper and iron metabolism in *S. pombe *similar to that in *S. cerevisiae*. This differs from previous data, which suggested that *frp1*, as well as *fip1 *and *fio1*, were repressed in a Cuf1p-dependent manner [43] (Figure 6a). This repression has been proposed to occur by direct binding of Cuf1p to TTTGTC motifs in the promoter region of these genes, as suggested by the observation that iron metabolism genes are induced in *cuf1*-Δ mutants, as well as by mutagenesis studies of the TTTGTC motifs [43]. Although these findings seem contradictory, the currently established role of Cuf1p as a transcriptional activator of high affinity copper transporters [38,49], together with the identification of DNA motifs that confer Fep1p regulation on the iron metabolism genes [42], now allow for an alternative interpretation of the previous results and are consistent with our findings.

The CuSE elements (GCTGA/T) that confer Cuf1p dependent activation for *ctr4*, *ctr5*, and *ctr6 *[49] are different from the TTTGTC motifs that are believed to be responsible for copper mediated repression of iron metabolism genes. Instead, the CuSEs identified in *S. pombe *are similar to the *S. cerevisiae *Ace1p binding motifs [73]. Moreover, the DNA binding domain of Cuf1p closely resembles that of Ace1p in *S. cerevisiae*, and a chimerical Cuf1p protein with an Ace1p DNA binding domain can complement a *cuf1*-Δ null mutant [38]. In the absence of additional DNA binding domains, it seems unlikely that Cuf1p would bind both the CuSE and TTTGTC motifs during copper starvation and simultaneously function as a transcriptional activator and repressor.

**Figure 7:**
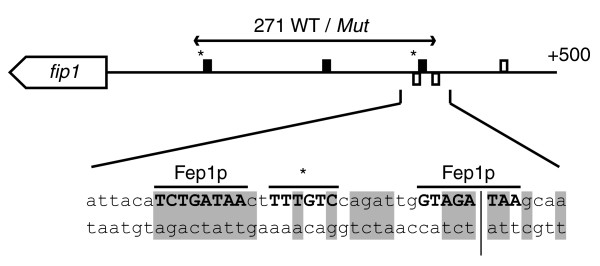
Proximity of TTTGTC motifs to Fep1p binding sites in the *fip1 *promoter. The 500 base pair (bp) region upstream of the *fip1 *open reading frame contains three TTTGTC motifs (closed rectangles) [43] and two Fep1p binding sites (open rectangles) [42]. A third Fep1p binding motif is found further upstream, but its role in Fep1p mediated regulation is unknown. Horizontal arrows indicate the 271 bp promoter fragment used previously [43] to study the effects of mutations in two of the TTTGTC motifs (asterisks). The area encompassing the two known Fep1p binding sites and the most distal mutated TTTGTC motif is shown enlarged to illustrate their proximity. Sequence residues that are conserved between the *fip1 *and *frp1 *promoters are shaded gray [42]. The vertical separator indicates the 3' end of the 271 bp promoter fragment.

The proposed role of the TTTGTC motifs in negative regulation of iron metabolism genes has been further supported by mutagenesis studies in a 271 base pair fragment of the *fip1 *promoter [43]. Mutations in two out of three TTTGTC motifs encompassed by this fragment abolished the apparent copper dependent repression of a reporter construct. Interestingly, a re-examination of this promoter fragment reveals that one of the TTTGTC motifs is located between two Fep1p binding sites, and mutation of this motif also alters two residues that are conserved between the Fep1p motifs in the *fip1 *and *frp1 *promoters (Figure 7). It is therefore likely that the induction of the reporter construct in the mutant resulted from the loss of Fep1p rather than Cuf1p regulation.

Taken together, the data above argue against a role for Cuf1p in repression of the iron metabolism genes *frp1*, *fip1*, and *fio1*. Instead, the minor induction of the iron uptake system during copper deprivation (as identified in the present study) and the strong induction in *cuf1*-Δ mutants [43] (Figure 3) points to a link between copper and iron metabolism similar to that in *S. cerevisiae*, in which copper starvation leads to delayed induction of the complete iron regulon as a result of a secondary iron starvation [19]. It is tempting to speculate that *frp1 *is also directly involved in copper metabolism, by binding of Cuf1p to the CuSE elements identified in its promoter region (Figure 3). This model would be in accordance with the situation in *S. cerevisiae*, in which copper uptake by the Ctr1p and Ctr3p transporters depends on the Frp1p homologs Fre1p and Fre2p [7,8], both of which are induced as part of the copper regulon [7,8,20,21]. Although other ferric reductase homologs exist in *S. pombe*, none of these are induced upon copper starvation. An updated model for Cuf1p and Fep1p regulation in *S. pombe*, including novel validated target genes, is presented in Figure 6b. The relative minor induction of the iron uptake system during the time of copper depletion assayed here could indicate that *S. pombe *contains larger iron stores than does *S. cerevisiae*, because the copper and iron concentrations in the synthetic media used to grow the two yeasts were identical. Alternatively, it could reflect a reduced demand for iron in *S. pombe *compared with *S. cerevisiae*, perhaps linked to the slower growth rate of the former.

The global transcriptional response to excess copper in *S. pombe *differs greatly from the highly specific detoxification mechanism adopted by *S. cerevisiae *[19] (Figure 2c). The limited response favored by *S. cerevisiae *is more efficient and conserves energy that would otherwise be used to synthesize the large number of proteins needed to protect the cell from oxidative stress. Interestingly, the response of budding yeast to other stressors such as hydrogen peroxide and heat shock results in the induction of a global stress response similar to that in *S. pombe *[48,69]. The difference in the characteristics of copper response in *S. cerevisiae *is in accordance with the belief that it uniquely evolved to deal with high levels of copper found in its environment.

A remaining question is how is the *S. pombe *response to excess copper regulated? Fission yeast utilizes several mechanisms for the sequestration of heavy metals, such as phytochelatin synthase, glutathione synthesis, and the metallothionein Zym1p. Although a previous study suggested that the role of Zym1p is limited to zinc metabolism [46], expression of its gene is increased in response to a number of other stresses as well [47], and we also found its induction to be more than twofold greater in high copper conditions (Figure 1). In contrast to budding yeast, however, we found that these detoxification systems cannot prevent the upregulation of a large number of additional stress response genes (Table 1 and Figure 2c).

There are two possible explanations for this difference. One is that the response to copper is nonspecific and purely regulated by general mechanisms that indirectly sense the effects of copper toxicity such as oxidative stress. An alternative explanation is that a specific response to detoxify excess copper exists, but it is not sufficient to prevent oxidative damage, resulting in an additional stress response. A number of observations suggest that the former model is more likely. First, the response to excess copper is similar to cadmium stress, suggesting that they are triggered by the same mechanism. A large number of the differentially expressed genes are known to be regulated by Sty1p and Atf1p, both of which are responsive to a wide variety of stressors [47], arguing against a unique role for copper. Further evidence comes from the timing of the transient response to low levels of copper, which is consistent with the response to other *S. pombe *stresses [47] and does not appear to be preceded by any specific response (Figure 2c). Two predicted glutathione *S*-transferases (SPAC688.04c and SPCC965.07c), as well as *zym1*, are upregulated simultaneously with other stress response genes, suggesting that putative metal scavenging systems are induced as part of the same response. Phytochelatin synthase expression could not reliably be measured on the arrays and experiments are currently underway to determine whether it is also induced together with the rest of the stress response. Our finding of a small number of genes specifically repressed during copper stress shows that some elements of the response may still be unique to copper, although regulation in these genes may be related to the toxic effects of copper rather than a copper dependent transcription factor.

Concentrations of free copper in *S. cerevisiae *are normally kept at less than one atom per cell [74]. In the absence of metallothionein expression, the buffering capacity for copper is greatly reduced, raising intracellular copper levels and causing toxicity. Interestingly, we found that *ace1*-Δ mutants subjected to excess copper do not induce a common environmental stress response, which is normally found when budding yeast is faced with an oxidative stress such as hydrogen peroxide [48,69]. This suggests that *S. cerevisiae *is unable to mount an alternative response to compensate for the oxidative stress triggered by copper in the absence of *ACE1*, giving a possible explanation for the previously described hypersensitivity to copper in these mutants [68]. Alternatively, the toxic effects of copper may be mediated by other mechanisms, such as displacement of similar trace metals (for example, zinc) from their physiologic binding sites, which become apparent before copper levels are sufficiently high to cause free radical stress. One way to test this would be to check whether the oxidative response to a small dose of hydrogen peroxide reduces copper hypersensitivity in *ace1*-Δ mutants. The phenomenon that a moderate response to one stressor can provide increased resistance to other types of stress is known as cross-protection [75,76]. Cross protection between copper or hydrogen peroxide induced stresses is only expected to occur if oxidative damage is the main reason for copper toxicity.

## Conclusion

Our comparisons between budding and fission yeast reveal that their considerable evolutionary distance has resulted in substantial differences in the regulation of copper and iron homeostasis. Despite these differences, the regulation of a core set of genes involved in the uptake of these metals remains conserved and provides valuable clues to key features of metal metabolism, as demonstrated by the putative regulation of *frp1 *by copper and iron in both yeasts. Genome wide comparisons are therefore useful to gain insight into the extent of conserved mechanisms between different species and can help to reveal the plasticity and adaptation of different aspects of cellular physiology.

## Materials and methods

### Strains, culture conditions and RNA isolation

For the experiments in *S. pombe*, we used the wild-type strain *972 h*-, as well as the *leu1-32 h*- pREP3X-*CUP1 *strain, which over-expresses the *S. cerevisiae CUP1 *metallothionein gene from a plasmid under the regulatable *nmt1 *promoter [77]. The over-expression strain was constructed in this study using a pair of specific primers (CUP1-FWD: 5'-CTCGAGATGTTCAGCGAATTA-3'; and CUP1-REV: 5'-CGTTTCATTTCCCAGAGCAGC-3') and a two-step cloning procedure, as previously described [78]. Standard methods [79] were used to culture *S. pombe *cells in liquid Edinburgh minimal medium (EMM) at 30°C, with shaking at 170 rpm.

For validating the putative *S. pombe *Cuf1p and Fep1p targets, we used the following strains: wild-type FY435 (*h*^+ ^*his7-366 leu1-32 ura4*-Δ*18 ade6-M210*), the *cuf1*-Δ disruption strain (*h*^+ ^*his7-366 leu1-32 ura4*-Δ*18 ade6-M210 cuf11*Δ*::ura4*), and the *fep1*-Δ disruption strain (*h*^+ ^*his7-366 leu1-32 ura4*-Δ*18 ade6-M210 fep1*Δ*::ura4*). All strains were grown in YE at 30°C.

The *S. cerevisiae ace1*-Δ mutant (*MATa*; *met15*; *ura3*; *his3 1*; *leu2*) was obtained from the *Saccharomyces *deletion project (Research Genetics, part of Invitrogen, Carlsbad, CA, USA) [80] and grown in synthetic complete (SC) medium supplemented with 2% glucose (Qbiogene, Irvine, CA, USA). Basal copper (CuSO_4_) and iron (FeCl_2_) levels were identical in both media, as defined by the manufacturer.

### Experimental design

Wild-type *S. pombe *was grown to an optical density (600 nm) of 0.15 to 0.2 before adding the stimulus, after which samples were collected at regular intervals, depending on the experiment. Cells were harvested by filtration and pellets immediately frozen on dry ice.

The high copper experiment in *S. pombe *was performed three times independently, using different final concentrations of CuSO_4_, namely 2, 10 and 25 μmol/l, and collecting at 0, 15, 30, 60, and 120 min. Conditions of low copper or low iron were induced, respectively, by either adding the copper chelator BCS (Sigma) to a final concentration of 100 μmol/l or the iron chelator ferrozine (Sigma, St. Louis, MO, USA) to a concentration of 300 μmol/l. The low copper experiment was independently repeated three times, whereas the low iron experiment was performed once. All samples were hybridized onto microarrays together with a reference from untreated wild-type cells from the same experiment (time 0).

The *S. pombe cuf1*-Δ and *fep1*-Δ experiments were performed twice independently, testing the following experimental conditions: wild-type FY435 untreated, wild-type FY435 100 μmol/l BCS and wild-type FY435 300 μmol/l ferrozine; *cuf1*-Δ untreated and *cuf1*-Δ 100 μmol/l BCS; and *fep1*-Δ untreated and *fep1*-Δ 300 μmol/l ferrozine. Cells were harvested 180 min after stimulus addition together with the untreated samples grown in parallel.

For the *CUP1 *over-expression experiment in *S. pombe*, cells were grown for 2 days in EMM without thiamine for steady-state induction of the *nmt1 *promoter. CuSO_4 _was added to a final concentration of 2 μmol/l, and cells were collected after 30 min. Cells carrying the pREP3X control vector were treated in the same way and used as reference. This experiment was performed once.

*S. cerevisiae ace1*-Δ mutants were exposed to high copper by adding CuSO_4 _(Sigma) to a final concentration of 8 μmol/l, and cells were collected at 0, 15, 60, 120, 180 and 240 min after addition, at an OD_600 _of 0.5 (corresponding to a mid-log growth phase). The experimental setup and concentrations were chosen according to the methods of van Bakel and coworkers [19] to facilitate comparison of expression profiles between the two studies. Samples for microarray analysis were harvested by centrifuging at 2000 *g *for 3 min, followed by snap freezing in liquid nitrogen.

### cDNA labeling, microarray hybridization, and data acquisition

For *S. pombe*, total RNA was isolated from all experimental and reference samples using a hot phenol protocol, as previously described [81,82]. Between 10 and 20 μg total RNA were labeled by direct incorporation of either fluorescent Cy3-dCTP or Cy5-dCTP (GE Healthcare, Chalfont St. Giles, Buckinghamshire, UK), and the fluorescently labeled product hybridized to *S. pombe *cDNA microarrays, as previously described [81]. Microarrays were subsequently scanned using a GenePix 4000B laser scanner (Molecular Devices, Sunnyvale, CA, USA) and fluorescence intensity ratios calculated with GenePix Pro (Molecular Devices, Sunnyvale, CA, USA).

For *S. cerevisiae*, total RNA isolation, cDNA synthesis and labeling, microarray production, and hybridization was done as described previously [83]. For each sample, 300 ng cDNA (with a specific activity of 2% to 4% dye-labeled nucleosides) was hybridized for 16 to 20 hours at 42°C. Microarray probes consisted of 70-mer oligonucleotides, and included 3,000 control features and duplicate probes for 6357 *S. cerevisiae *genes. Slides were scanned in an Agilent DNA Microarray Scanner (model G2565BA; Agilent Technologies, Santa Clara, CA, USA). Spot quantification was carried out using Imagene 4.0 (Biodiscovery, El Segundo, CA, USA).

The entire raw dataset is available from the ArrayExpress database [84], accession number E-TABM-120.

### Microarray data analysis

Normalization of the *S. pombe *microarray data was performed using an in-house script [81]. The *S. cerevisiae *data were normalized by applying a Lowess function per subgrid on all gene spots [85], using the marrayNorm R package v1.1.3 [86]. Genes for which more than 50% of data points were missing were discarded from further analysis in both datasets.

Genes were considered differentially expressed in the high copper experiment when they exhibited a greater than 1.5-fold change at at least one time point after stimulation with 2 μmol/l CuSO_4 _and at least a similar change in response to 25 μmol/l CuSO_4_. In the low copper experiment, genes induced or repressed by more than 1.5-fold at at least one time point in two out of three biologic repeats were selected. Similar cutoffs were used for the low iron (300 μmol/l ferrozine) and the high copper (8 μmol/l CuSO_4_) experiments in *S. cerevisiae ace1*-Δ mutants, selecting genes that were induced or repressed by at least 1.5-fold at one or more time points. Hierarchical clustering was done in Genespring 6.1 (Agilent Technologies, Santa Clara, CA, USA). Genes were assigned to functional classes according to the Gene Ontology consortium database [87].

### Comparison with data from *S. cerevisiae*

Raw data and lists of *S. cerevisiae *copper regulated genes were obtained from the report by Van Bakel and coworkers [19]. Genes with a prospective *S. pombe *ortholog were determined using a table of curated orthologs as previously described [88] or by best reciprocal hit BLAST analysis [89]. The total number of curated orthologs available at the time of the analysis was 3,655. If an *S. pombe *ortholog to an *S. cerevisiae *gene could not be identified based on the above criteria, we instead used the BLAST algorithm [89] to select the most similar *S. pombe *sequences for our interspecies comparisons of copper metabolism. The best matching hits in *S. pombe *with an e value less than 1 × e^-20 ^and a minimum match length of 80% were selected as putative functional homologs.

### Analysis of transcription factor binding motifs

DNA regulatory patterns were derived from manual alignments of experimentally confirmed binding motifs and determined as KYWGATAW (K = G/T, Y = C/T, and W = A/T) for Fep1p [41,42] and WNNNGCTGD (W = A/T, N = any, and D = G/A/T) for Cuf1p [36,37,90]. These patterns were subsequently used to search for putative novel binding sites in the 800 base pair region upstream of the transcriptional start site of genes induced in low copper or low iron conditions. When necessary, upstream regions were truncated to prevent overlap with other open reading frames. Both the retrieval of upstream sequences and pattern matching were done at the Regulatory Sequence Analysis Tools website [91].

### Quantitative real-time PCR

Putative novel *S. pombe *targets for Cuf1p and Fep1p were validated by quantitative real-time PCR in *cuf1*-Δ and *fep1*-Δ disruption strains. Total RNA was isolated as previously described [81], treated with Turbo DNase (Ambion, Foster City, CA, USA) in order to remove any genomic DNA contamination, and reverse transcribed using random hexamers and Omniscript RT Kit (Qiagen, Venlo, Limburg, The Netherlands), in accordance with the manufacturer's instructions. Expression levels were quantified by using SYBR GreenER qPCR Supermix ABI PRISM (Invitrogen) on an Applied Biosystems (Foster City, CA, USA) 7300 Real-Time PCR System and normalized using *act1 *expression levels as a reference. Primer sequences used are available as supplementary data (Additional data file 2 [Supplementary table 5]).

## Additional data files

The following additional data are available with the online version of this paper. Additional data file 1 includes tables providing details on all genes induced or repressed in the experimental conditions assayed here. Additional data file 2 includes a table summarizing the sequences of all qPCR primers used.

## Supplementary Material

Additional data file 1 Provided are tables presenting details on all genes induced or repressed in the experimental conditions assayed here. Click here for file

Additional data file 2 Provided is a table summarizing the sequences of all qPCR primers used. Click here for file
