# Supplementary material for: Global transcriptional responses of fission and budding yeast to changes in copper and iron levels: a comparative study
Source: Genome Biol. 2007 May 3;8(5):R73. doi: 10.1186/gb-2007-8-5-r73 (PMC1929147; doi:10.1186/gb-2007-8-5-r73)
Supplement: Additional data file 1 — Provided are tables presenting details on all genes induced or repressed in the experimental conditions assayed here. [file gb-2007-8-5-r73-S1.pdf]

Supplementary Table 1A. Genes induced in *S. pombe* copper starvation (100 μM BCS)

Gene Name	Max Cu-	Max Cu+	Max Fe-	Annotation
**Metal ion transport**				
ctr4 [a]	24.4*	1.0	-1.2	copper transporter
ctr5 [a]	6.5	-1.2	4.3	copper transporter
ctr6 [a]	1.9	-1.1	1.5	copper transporter
str1 [a]	1.6	1.8	1.6	siderochrome-iron transporter
frp1 [a]	1.8	18.1	16.9	Ferric reductase
**Other transport**				
SPBC887.17 [a]	1.5	-1.4	1.0	uracil permease
**Peroxisomal proteins**				
pex7 [a]	2.3	-1.1	1.6	peroxisomal targeting signal receptor
SPAC3G6.05 [a]	2.3	1.3	2.4	Mvp17/PMP22 family, localisation peroxisomal membrane
**Others**				
SPAC458.03 [a]	1.5	-1.1	-1.1	leucine-rich repeat protein involved in telomere maintenance
SPAPB18E9.02	1.6	1.2	-1.1	serine/threonine protein kinase
SPBPB2B2.05 [a]	2.2	1.1	1.5	GMP synthase

* Value obtained by quantitative real-time PCR
[a] Contains putative Cufp1p binding site in the promotor region

Supplementary Table 1B. Genes repressed in *S. pombe* copper starvation (100 μM BCS)

Gene Name	Max Cu-	Max Cu+	Max Fe-	Annotation
**Flavoproteins**				
obr1	-1.7	1.6	1.2	flavodoxin
SPAC869.02c	-1.9	1.3	-1.4	flavoprotein
**Oxidoreductases and dehydrogenase**				
SPAC26H5.09c	-1.3	1.1	-1.1	oxidoreductase
SPAC750.01	-1.6	1.9	-1.2	pseudogene, oxidoreductase
SPBC23G7.10c	-1.6	2.7	1.3	NADH-dependent flavin oxidoreductase
**Antioxidants**				
gst2	-1.5	3.0	-1.6	glutathione S-transferase
sod1	-2.2	1.7	-1.6	Cu, Zn-superoxide dismutase

Supplementary Table 2A. Genes induced in *S. pombe* iron starvation (300 μM Ferrozine)

Gene Name	Max Cu-	Max Cu+	Max Fe-	Annotation
**Metal ion transport**				
ctr5	6.5	-1.2	4.3	copper transporter
frp1 [a]	1.8	18.1	16.9	ferric-chelate reductase activity
fip1 [a]	1.8	2.9	2.4	iron permease
fio1 [a]	-1.1	NA	2.6	iron transport multicopper oxidase
str3 [a]	1.2	4.1	6.8	siderochrome-iron transporter Str3
str1 [a]	1.6	1.8	1.6	siderochrome-iron transporter Str1
SPBC947.05c [a]	1.1	-1.1	4.3	ferric-chelate reductase activity
SPAC8C9.12c	-1.6	1.8	2	mitochondrial carrier, putatively involved in iron transport
**Other transport**				
vht1	1.9	-2.1	1.9	vitamin H transporter
vps53 [a]	-1.0	2.0	2.0	involved in intracellular protein transport
**Iron-sulfur cluster assembly**				
isu1 [a]	1.2	1.4	1.6	iron-sulfur cluster assembly scaffold protein
**Peptide biosynthesis**				
sib1 [a]	1.4	2.1	2.2	ferrichrome synthetase
sib2 [a]	1.3	3.2	3.2	ornithine N5 monooxygenase, involved in iron homeostasis
**Others**				
SPAC1F8.02c [a]	-1.3	NA	15.5	GPI-anchored glycoprotein
ppr1 [a]	1.5	-2.1	2.6	L-azetidine-2-carboxylic acid acetyltransferase
SPAC22F8.05	-1.3	2.6	1.6	alpha,alpha-trehalose-phosphate synthase
tps1	-1.2	2.4	1.9	alpha,alpha-trehalose-phosphate synthase [UDP-forming]
rds1 [a]	-1.8	3.1	1.9	involved in response to stress
ish1 [a]	-2	2.2	1.8	LEA domain protein
srx1 [a]	-5.3	1.4	1.6	Sulphiredoxin
SPAC3G6.05	2.3	1.3	2.4	Mvp17/PMP22 family
SPAC56E4.03 [a]	1.2	1.6	1.7	aromatic aminotransferase
SPBC1271.07c	-1.4	2.0	1.8	N-acetyltransferase
sid4 [a]	NA	NA	1.6	SIN component
SPBC27B12.03c [a]	1.4	1.7	2.1	lathosterol oxidase, uses iron as cofactor
cfh4	-1.2	1.1	1.5	Chs Four Homologue, cell wall chitin biosynthesis
SPCC70.08c	1.7	2.8	3.1	SAM dependent methyltransferase
**Unknown**				
SPAC23H3.15c [a]	-3.6	6.6	1.7	
SPAC29A4.17c	-1.2	NA	1.6	
SPBC1271.08c [a]	-1.1	1.8	2.4	
SPBC21C3.19	-2.7	2.7	2.7	
SPAC15E1.02c [a]	-1.6	1.2	1.6	

[a] Contains putative Fep1p binding site in the promotor region

Supplementary Table 2B. Genes repressed in *S. pombe* iron starvation (300 μM Ferrozine)

Gene Name	Max Cu-	Max Cu+	Max Fe-	Annotation
**Metal metabolism**				
fep1	1.6	NA	-1.9	iron-sensing transcriptional regulator
hem3	2.0	NA	-2.0	hydroxymethylbilane synthase
**Localized to the mitochondrion**				
cyc1	1.6	1.1	-2.9	cytochrome c
sdh3	1.6	-1.3	-2.2	succinate dehydrogenase
sdh2	1.9	-1.3	-2.9	succinate dehydrogenase
SPAC20G8.04c	1.6	1.6	-1.8	electron transfer flavoprotein-ubiquinone oxidoreductase
SPAP14E8.05c	1.2	1.1	-2.2	conserved eukaryotic protein
tim18	1.7	NA	-2.9	membrane anchor subunit
**Transporters**				
bsu1	-1.1	NA	-3.0	MFS efflux transporter
SPAC869.05c	-1.3	4.1	-2.0	sulfate transporter
SPBC13A2.04c	-2.1	-7.1	-2.3	PTR family peptide transporter
**Iron/sulfur cluster proteins**				
leu2	1.4	-1.3	-1.6	3-isopropylmalate dehydratase
SPAC24C9.06c	1.8	1.7	-1.6	aconitate hydratase
**Thiamine biosynthesis**				
SPBP8B7.18c	1.3	-1.3	-1.9	TENA/THI domain
nmt1	-1.2	-1.5	-2.3	repressed by thiamine
**Others**				
pho4	-1.2	-1.2	-1.8	acid phosphatase activity
rpl4302	1.2	NA	-1.6	60S ribosomal protein L37a
SPCC162.03	1.1	-1.1	-1.5	short chain dehydrogenase
gln1	1.4	-1.9	-2.1	glutamate-ammonia ligase, glutamine biosynthesis
**Unknown**				
SPAC17G8.08c	1.5	-1.3	-1.9	
SPBC1289.14	NA	NA	-2.5	
SPBC29A3.21	1.5	NA	-2.4	
SPBC530.07c	1.2	-1.2	-2.0	
SPAC694.04c	1.2	-1.3	-1.6	

Supplementary Table 3A. Genes induced in *S. pombe* copper excess (2.0 μM $CuSO_4$)

Gene Name	Max Cu-	Max Cu+	Max Fe-	Annotation
**Antioxidants**				
SPAC688.04c[a]	-1.3	1.9	1.4	glutathione S-transferase
SPCC965.07c[a]	-1.5	3.0	-1.6	glutathione S-transferase
pmp20[a]	1.2	1.7	1.2	peroxiredoxin, AhpC/TSA family
sod1[a]	-2.2	1.7	-1.6	Cu, Zn superoxide dismutase
trx2[a]	1.3	1.9	1.2	thioredoxin I
zym1[a]	-1.9	1.8	1.4	zinc metallothionein
**Carbohydrate metabolism**				
SPBC32F12.10	1.4	1.8	1.3	phosphoglucomutase involved in glucose 1-phosphate and 6-phosphate utilization
SPCC663.09c	1.1	1.7	1.1	short chain dehydrogenase
tps1[a]	-1.2	2.4	1.9	alpha, alpha-trehalose-phosphate synthase
zwf1 [a, b]	1.3	2.4	1.3	glucose-6-phosphate 1-dehydrogenase involved in pentose-phosphate pathway
**Lipid biosynthesis**				
SPAC26F1.04c[a]	-1.4	2.4	1.3	zinc binding dehydrogenase
SPBC27B12.03c	1.4	1.7	2.1	lathosterol oxidase, sterol desaturase
**Iron uptake**				
fip1	1.8	2.9	2.4	iron permease
frp1	1.8	18.1	16.9	ferric-chelate reductase activity, involved in iron ion transport
str3	1.2	4.1	6.8	membrane transporter involved in iron-siderochrome transport
**Peptide biosynthesis**				
sib1	1.4	2.1	2.2	ferrichrome synthetase
sib2[a]	1.3	3.2	3.2	ornithine N5 monooxygenase, involved in iron homeostasis
**Protein folding / chaperone**				
SPBC1711.08[b]	1.2	1.9	1.4	chaperone activator, target Hsp90p
SPBC4F6.16c[a,b]	-1.2	1.8	1.3	disulfide oxidoreductase, FAD dependent, involved in protein disulfide bond formation
hsp16 [a, b]	-1.3	23.3	1.5	heat shock protein 16
hsp9 [a, b]	-1.7	3.3	1.5	heat shock protein 9
isp6 [a, b]	1.3	2.1	1.4	serine protease, involved in sexual differentiation
psi[a, b]	1.3	3.8	1.6	Psi protein, heat shock protein
Hsp70[b]	1.4	1.9	1.4	heat shock protein 70 family
sti1[a, b]	1.3	2.5	1.3	chaperone activator
swo1: hsp90[b]	1.5	2.0	1.4	heat shock protein, hsp90 family
ubi4	1.2	2.6	-1.1	ubiquitin, involved in meiosis
ubx3 [a, b]	1.1	1.9	1.2	UBX domain protein
wos2	1.4	2.5	1.4	chaperone activator, interacts with Hsp90p
**Signaling and transcriptional regulation**				
ish1[a]	-2	2.2	1.8	LEA domain protein, regulated by Atf1p, MAPK pathway
srk1[a]	-1.9	2.3	1.4	MAPK-activated protein kinase, Sty1 SAPK cascade
**Stress response**				
cdc48[b]	1.4	1.8	1.2	AAA family ATPase
cuf5[a]	NA	NA	NA	MFS multidrug efflux transporter
rds1[a]	-1.8	3.1	1.9	adenine-repressible; regulated by glucose, ammonium, phosphate, CO_2 and temp.
slt1[a]	-1.2	5.9	1.6	involved in response to drug and caffeine
**Other**				
cgs1[a]	-1.4	1.7	1.3	cAMP-dependent protein kinase, negative regulator of meiosis
SPBC12C2.03c	-1.3	1.7	1.1	FAD binding oxidoreductase
SPAC19G12.09	1.8	2.8	1.4	oxidoreductase , aldo/keto reductase
SPBC215.11c[a]	1.6	2.0	1.2	aldo/keto reductase
SPCC285.01c [b]	1.3	2.3	1.2	quinone oxidoreductase
SPBC23G7.10c[a]	-1.6	2.7	1.3	NADH-dependent flavin oxidoreductase
SPBC725.10[a]	-1.4	3.0	1.3	similar to peripheral-type benzodiazepine receptor
SPAC977.14c	-1.5	1.9	-1.2	aldo/keto reductase
Plr[a]	1.5	2.5	1.3	pyridoxal reductase, aldo/keto reductase family 8
vip1[a]	-1.2	2.2	1.3	protein with RNA recognition motif
wis2	1.4	2.0	1.4	cyclophilin, involved in mitosis regulation
SPBC359.05[b]	1.4	2.0	1.7	ABC transporter, MDR subfamily, unknown specificity

**Sulphur amino acid biosynthesis**				
SPAC869.05c	-1.3	4.1	-2.0	sulfate transporter activity, involved in sulfate transport
SPBPB10D8.01	1.3	2.4	-2.1	membrane transporter, unknown specificity
SPBP16F5.08c	1.2	2.7	-1.2	flavin dependent monooxygenase, involved in disulphide bond formation
pof1	1.2	2.3	-1.1	F-box protein
SPCC1739.06c	-1.6	2.8	-1.1	uroporphyrin methyltransferase
SPAPB24D3.08c[ab]	1.6	1.7	1.1	NADP-dependent oxidoreductase
**Unknown function**				
SPBC11C11.06c[a]	-1.6	1.7	1.6	
SPBC16E9.16c[a]	-2.5	3.9	1.5	
SPBC17D11.03c	-1.1	1.9	-1.1	
SPCC191.01[a]	-1.4	3.2	1.5	
SPBC21B10.08c	1.2	2.2	1.3	
SPBC21C3.19[a]	-2.7	2.7	2.7	
SPAC22F3.11c	1.2	1.7	-1.1	
SPAC25H1.01c[a]	-3.6	6.6	1.7	
SPBC3B9.01[b]	1.4	5.0	1.6	
SPCC576.04[a]	-1.4	1.9	1.4	
SPCC63.14[a]	1.2	2.1	1.3	
SPBC660.06[a]	1.3	2.9	1.4	
SPBC725.03[a]	-2.2	1.8	NA	
SPAC750.01[a]	-1.5	1.9	-1.2	
SPACUNK4.15[a]	1.3	1.8	1.4	
SPBP35G2.02	-1.2	1.6	1.2	

a) Core Environmental Stress Response Genes

b) Genes with orthologs in *S. cerevisiae* that show similar expression changes when *S. cerevisiae ace1*-Δ mutants are exposed to 8 μM $CuSO_4$

Supplementary Table 3B. Genes repressed in *S.pombe* copper excess (2.0 μM $CuSO_4$)

Gene Name	Max Cu-	Max Cu+	Max Fe-	Annotation
**Aminoacid metabolism and transport**				
SPAC1039.01	-1.4	-2.2	-1.5	amino acid permease family
SPAC1039.08	-1.1	-3.0	-1.3	serine acetyltransferase, involved in cysteine metabolism
SPBC359.03c	1.1	-1.5	-1.2	amino acid permease family
SPAP7G5.06	-1.4	-2.6	-1.5	amino acid permease family
**Ribosomal proteins**				
rpl44: rpl28[a]	1.3	-1.6	-1.3	60S ribosomal protein L28/L44
rps20[a]	1.2	-1.5	-1.5	40S ribosomal protein S20
**Transporters**				
SPAC11D3.18c	1.7	-6.5	-1.8	membrane transporter, unknown specificity
SPAC1399.02	1.5	-1.6	-1.4	membrane transporter, unknown specificity
SPAC212.10	1.1	-2.0	-1.2	pseudo-malic acid transport protein
SPBC8E4.01c[b]	1.3	-1.8	1.3	inorganic phosphate transporter
ght6: meu12	1.4	-1.5	-1.1	hexose transporter
isp4	-1.4	-2.0	-1.1	OPT oligopeptide transporter
ptr2	-2.1	-7.1	-2.3	PTR family peptide transporter, transloctes di-/tripeptides
**Others**				
SPAC1039.02	1.3	-2.0	-1.3	calcineurin-like phosphoesterase
SPAC11D3.15	1.4	-4.0	-1.5	oxoprolinase
SPCC1223.09	-1.1	-2.6	-1.3	Uricase
SPCC132.04c	1.9	-1.6	2.6	glutamate dehydrogenase NAD-specific, involved in nitrogen & glutamate metabolism
SPCC222.11	1.1	-1.7	-1.2	coproporphyrinogen III oxidase
gln1[a]	1.4	-1.9	-2.1	glutamate-ammonia ligase, involved in glutamate and nitrogen metabolism
mei2	1.3	-1.9	-1.6	RNA-binding protein, involved in meiosis
SPBPB2B2.06c	1.2	-1.9	1.3	calcineurin-like phosphoesterase
SPBPB7E8.01	-2.1	-1.7	1.5	glycoprotein
prl3	1.9	-2.0	-1.2	non-coding RNA
**Unknown function**				
SPAC5H10.01	1.9	-18	-1.5	

a) Core Environmental Stress Response Genes

b) Genes with orthologs in *S. cerevisiae* that show similar expression changes when *S. cerevisiae ace1*-Δ mutants are exposed to 8 μM $CuSO_4$

Supplementary Table 4A. Genes induced in copper excess in *S. cerevisiae ace1-Δ* mutants (8.0 μM $CuSO_4$)

Gene Name	Max Cu+	Annotation
**protein catabolism**		
MDJ1	3.5	Protein involved in mitochondrial biogenesis and protein folding during heat shock
YDJ1	3.6	Protein involved in protein import into mitochondria and ER, homolog of E. coli DnaJ
PRE3	2.0	Proteasome subunit beta1_sc involved in activity of the proteasome
LAP4[a]	2.0	Aminopeptidase I (yscI, API) of the vacuole
RPN2	1.9	Non-ATPase component of 26S proteasome complex
PRE2	2.0	Proteasome subunit beta5_sc (protease yscE subunit Pre2/Prg1)
RPN7	2.0	Non-ATPase of the 26S proteasome complex
RPT5	2.4	ATPase component of 26S proteasome complex
RPT4	1.8	Component of the 19S regulatory cap complex of the 26S proteasome complex
PRE10	2.2	Proteasome subunit alpha7_sc (YC1 - protease yscE subunit 1)
PRE8	1.6	Proteasome subunit alpha2_sc (protease yscE subunit Y7)
PRE5	2.0	Proteasome subunit alpha6_sc
UBX4	1.8	Protein containing a UBX domain, which are found in ubiquitin regulatory proteins
DSK2	2.2	Protein involved in ubiquitin-dependent protein degradation and proteasome
RPN6	2.0	Non-ATPase subunit of the 26S proteasome complex
CDC48[b]	2.1	component of the ubiquitin/proteasomal-dependent ER-associated degradation system
RPT2	1.8	ATPase component of the 26S proteasome complex
RPN9	1.9	Non-ATPase subunit of the 26S proteasome complex
RPT3	2.0	ATPase component of the 26S proteasome complex
PRE6	2.2	Proteasome subunit alpha4_sc
UBC4	2.0	Ubiquitin-conjugating (E2) enzyme
PIM1	2.0	Serine protease required for intramitochondrial proteolysis
SHP1[b]	2.0	Potential regulatory subunit for Glc7p
PRD1	2.3	Proteinase yscD (saccharolysin), contains the zinc metalloendoprotease motif HEXXH
RPN4	2.7	Subunit of the regulatory particle of the proteasome
NPL4	2.3	Nuclear pore protein required for ubiquitin-dependent degradation of ER substrates
UMP1	2.1	Proteasome maturation factor, a chaperone involved in proteasome assembly
RPT6	1.8	ATPase component of 26S proteasome complex
SCL1	1.8	Proteasome subunit alpha1_sc (YC7alpha/Y8, protease yscE subunit 7)
RPN10	1.9	Non-ATPase component of the 26S proteasome complex
ECM29	3.0	Protein possibly involved in cell wall structure or biosynthesis
PUP3	1.8	Proteasome subunit beta3_sc
PRE1	2.2	Proteasome subunit beta4_sc (C11 - protease yscE subunit 11)
PRB1[a,b]	2.4	Protease B (yscB/PrB/cerevisin), serine protease of the subtilisin family
PRE4	1.8	Proteasome subunit beta7_sc
RPN12	2.1	Non-ATPase component of 26S proteasome complex
RPN11	2.0	Protein of the 19S regulatory particle of the proteasome
RPN3	1.9	Non-ATPase component of 26S proteasome complex
**response to stress**		
HSP42	6.1	Heat shock protein with similarity to Hsp26p, involved in restoration of the cytoskeleton
HSP78[a]	4.7	Mitochondrial heat shock protein of the ClpB family of ATP-dependent proteases
HSC82[b]	4.5	Chaperonin homologous to E. coli HtpG and mammalian HSP90
SSA4	11.7	Protein chaperone of the HSP70 family
AHA1[b]	3.8	Protein of unknown function
YAP1	2.0	Possible redox sensor involved in oxidative stress response
TSL1	2.0	Regulatory component of the trehalose-6-phosphate synthase/phosphatase complex
KAR2	6.2	Heat shock protein of the ER lumen
HSP104[a]	5.1	Heat shock protein
YJL144W	3.5	Protein of unknown function
HCH1	3.7	Protein of unknown function
UBC5	2.4	Ubiquitin-conjugating enzyme
SSA3	1.8	Chaperone of the HSP70 family
HSP26[a,b]	7.1	Heat shock protein of 26 kDa
YHB1	2.5	Flavohemoglobin involved in protection from nitrosative stress
TRX2[b]	2.0	Thioredoxin II
HSP82	5.8	Heat-inducible chaperonin homologous to E. coli HtpG and mammalian HSP90
GRE3	1.9	Aldo/keto reductase with NADPH specificity, induced by osmotic stress
HSP12[a,b]	2.6	Heat shock protein of 12 kDa, induced by heat, osmotic stress, and oxidative stress
MAG1[a]	2.9	DNA-3-methyladenine glycosylase, repair of alkylation-damaged DNA
STI1[b]	5.3	Stress-induced protein required for optimal growth at high and low temperature
SSA2	7.3	Cytoplasmic protein chaperone of the HSP70 family
HSP10	3.0	Mitochondrial chaperonin that cooperates with Hsp60p, counterpart of E. coli GroES
SSE1	4.5	Heat shock protein of the HSP70 family

SSE2	1.8	Heat shock protein of the HSP70 family
SSE2	1.8	Heat shock protein of the HSP70 family
**Transport**		
BTN2	17.9	Protein with a role in cellular pH homeostasis
SSA1[b]	6.9	Cytoplasmic chaperone and heat shock protein of the HSP70 family
GSF2	1.6	Protein involved in glucose repression
HSP60	2.5	Mitochondrial chaperonin that cooperates with Hsp10p, homolog of E. coli GroEL
YKT6	1.8	Synaptobrevin (v-SNARE) homolog essential for endoplasmic reticulum-Golgi transport
MIA40	1.9	Mitochondrial protein that may play a role in cell cycle progression
VPS65	2.0	Class F vacuolar sorting protein involved in Prc1p vacuolar trafficking pathway
SSC1	2.4	Mitochondrial protein that acts as an import motor with Tim44p and as a chaperonin
YCF1[b]	2.1	Vacuolar glutathione S-conjugate transporter; ATP-binding cassette (ABC) superfamily
ATG8[a]	2.0	Protein required for delivery of autophagic vesicles to the vacuole
GTS1	1.6	Putative zinc-finger transcription factor of the Gcs1p/Glo3p/Sps18p family
ERV29	1.9	Component of COPII-coated vesicles, required for vesicular transport
**Organelle organization and biogenesis**		
SED1	1.9	Abundant cell surface glycoprotein
TCP1	1.6	Component of chaperonin-containing T-complex (TCP ring complex, TRiC)
PNC1[a]	1.8	Pyrazinamidase and nicotinamidase
YHR138C[a]	2.1	Protein possibly involved in vacuolar fusion
**Protein modification**		
BET4	1.9	Geranylgeranyltransferase Type II alpha subunit, complexes with Bet2p
UBA1	2.1	Ubiquitin-activating (E1) enzyme
STE24	1.6	Prenyl-dependent protease involved both in the first N-terminal proteolytic step
ERO1[b]	3.2	Protein required for protein disulfide bond formation in the endoplasmic reticulum
RTS3	2.8	Protein of unknown function
YPI1[a]	1.8	Protein of unknown function, may physically interact with Glc7p and Ppz1p
**Protein biosynthesis**		
MEF2	1.8	Putative mitochondrial translation elongation factor G
DBP1	1.9	ATP-dependent RNA helicase of the DEAD box family
SIS1[b]	4.0	Protein required for initiation of translation; DnaJ family of protein chaperones
MRPL11	1.8	Mitochondrial ribosomal protein of the large subunit (YmL11)
FES1[b]	3.2	Protein involved in resistance to H2O2
**Transcription**		
MBF1	3.0	Transcriptional coactivator of Gcn4p
PDR1	1.7	Zinc-finger transcription factor; regulator of genes involved in multiple drug resistance
YOD1[a]	1.9	Protein of unknown function, has a single C2H2-type zinc finger
**Others / Unknown**		
EDC2	1.8	Protein involved in mRNA decapping
YOL155C	2.7	Protein with similarity to S. cerevisiae glucan 1,4-alpha-glucosidase
ISU2	2.6	Protein with similarity to iron-sulfur cluster nitrogen fixation proteins
GCV2	1.8	Glycine decarboxylase pyridoxal phosphate containing P subunit
ARO9	2.6	Aromatic amino acid aminotransferase II
FRM2	13.5	Protein involved in the integration of lipid signaling pathways with cellular homeostasis
CPR6	5.0	Cyclophilin (peptidylprolyl cis-trans isomerase or PPIase)
GSH1	1.7	gamma-Glutamylcysteine synthetase
GTT2	3.4	Glutathione transferase
HEM3	1.7	Porphobilinogen deaminase (pre-uroporphyrinogen synthase)
PDI1	2.0	Protein disulfide isomerase and oxidoreductase
YFL006W	1.8	Protein of unknown function
YFR024C	1.7	Protein with possible role in the regulation of actin cytoskeletal organization
YLR108C	2.7	Protein containing a K+ channel tetramerization domain
REH1	1.9	Protein possibly involved in proteasome function
YLR247C	1.9	Protein containing an SNF2 related N-terminal domain
RBF9	3.8	Protein with high similarity to S. cerevisiae Stf2p
YIL169C	2.3	Protein of unknown function
DRE2	2.5	Protein of unknown function
YKR011C	2.5	Protein of unknown function
SGT2	3.2	Protein of unknown function
OYE3[a]	2.1	NADPH dehydrogenase (old yellow enzyme), isoform 3
YMR034C	2.1	Protein of unknown function
YPR158W	4.0	Protein of unknown function
YML131W[b]	2.3	Putative NAD-dependent oxidoreductase
YMR155W	1.8	Protein of unknown function

YNL155W	2.9	Protein predicted to have structural similarity to disulfide oxidoreductases
YNL134C	2.1	Member of the zinc-containing alcohol dehydrogenase family
YOR052C[a]	2.4	Protein containing an AN1-like zinc finger domain
APJ1	2.5	Protein induced by cell stress
YDL206W	1.7	Putative membrane transporter of the Ca2+:cation antiporter (CaCA) family
YDR154C	1.9	Protein of unknown function
HSP31	2.5	Member of the DJ-1 or PfpI family, has high similarity to S. cerevisiae Ypl280p
YBR099C	3.1	Protein that might affect sensitivity to UV radiation
YCR061W	1.9	Protein that may be involved in response to heat and high salinity
YAR068W	1.9	Protein of unknown function
YHR214W-A	2.1	Protein of unknown function
FMP52	2.1	Protein of unknown function
YEL045C	1.7	Protein of unknown function
ZTA1[b]	1.7	Zeta-crystallin homolog
CPR1	1.8	Cyclophilin (peptidylprolyl cis-trans isomerase or PPIase) of the cytosol
ZWF1[b]	1.8	Glucose-6-phosphate dehydrogenase
ZPR1	2.1	Zinc finger protein required for normal cell proliferation

a) Core Environmental Stress Response Genes
b) Genes with orthologs in *S. pombe* that show similar expression changes when *S. pombe* is exposed to 2 μM $CuSO_4$

Supplementary Table 4B. Genes repressed in copper excess in *S. cerevisiae ace1-Δ* mutants (8.0 μM $CuSO_4$)

Gene Name	Max Cu+	Annotation
**Transport**		
TPO2	-2.2	Polyamine transport protein; multidrug-resistance 12-spanner (DHA12) family
CTR1	-2.3	Copper transport protein required for high-affinity uptake of copper
PIC2	-2.8	Member of the mitochondrial carrier (MCF) family of membrane transporters
PHO84[a,b]	-2.7	High-affinity inorganic phosphate/H+ symporter
HXT4	-2.0	Moderate- to low-affinity hexose transporter
TPO4	-1.8	Polyamine transport protein; multidrug-resistance 12-spanner (DHA12)
PDR5	-5.5	Drug-efflux pump involved in resistance to multiple drugs
OPT2	-3.1	Oligopeptide transporter
DIC1	-1.8	Mitochondrial dicarboxylate transport protein
AQY1	-2.1	Aquaporin water channel protein
FET4	-2.3	Low-affinity Fe(II) transport protein
MTH1	-1.9	Repressor of hexose transport genes
OAC1	-1.6	Mitochondrial oxaloacetate transporter
**Amino acid and derivative metabolism**		
MET12	-1.9	Methylenetetrahydrofolate reductase (MTHFR), involved in methionine biosynthesis
ECM17	-1.6	Sulfite reductase (ferredoxin)
CAR2	-1.9	Ornithine aminotransferase (ornithine oxo-acid aminotransferase)
SAM2	-2.1	S-adenosylmethionine synthetase 2
MET6	-2.4	Homocysteine methyltransferase; methionine synthase, cobalamin-independent
SER3	-2.7	3-phosphoglycerate dehydrogenase, catalyzes the first step in synthesis of serine
LYS4	-1.8	Homoaconitate hydratase (homoaconitase), third step in lysine biosynthesis pathway
ILV3[a]	-2.1	Dihydroxyacid dehydratase (DAD), third step in valine and isoleucine biosynthesis
LEU1	-2.3	3-Isopropylmalate dehydratase, second step in leucine biosynthesis pathway
BAT1	-1.7	Mitochondrial branched-chain amino acid transaminase
**Carbohydrate metabolism**		
GSY1	-2.3	UDP-glucose-starch glucosyltransferase (glycogen synthetase) isoform 1
HAP4	-2.2	Transcription factor with acidic activation domain
MDH2	-1.8	Malate dehydrogenase, cytosolic, probably functions in the glyoxylate cycle
KNH1	-1.8	Putative secreted protein with similarity to Kre9p
SDH2	-1.8	Succinate dehydrogenase (ubiquinone) iron-sulfur protein subunit
**response to stress**		
HSP30	-5.7	Heat shock protein located in the plasma membrane
GRE1	-1.7	Protein induced by osmotic stress
CCP1	-1.7	Cytochrome-c peroxidase, involved in destruction of toxic radicals in the cell
**lipid metabolism**		
SCS7	-2.1	Ceramide hydroxylase
SUR2	-1.7	Hydroxylase involved in sphingolipid metabolism
ATF2	-1.7	Sterol O-acetyltransferase

**transcription**		
ACE1	-12.8	Copper-dependent transcription factor
CUP9	-2	Homeodomain protein involved in copper homeostasis
**Others / Unknown**		
LDB7	-1.7	Protein of unknown function
SPI1	-2.4	Protein induced in stationary phase, has similarity to Sed1p
YER067W	-3.1	Protein of unknown function
YIR043C	-1.8	Member of the duplication (DUP) family
STP4	-3.3	Protein with strong similarity to Stp1p
YPR157W	-2.8	Protein with high similarity to S. cerevisiae Vps62p
YPL014W	-2.0	Protein of unknown function
FMP48	-2.0	Serine/threonine protein kinase of unknown function
ICY1	-2.0	Protein of unknown function
DLD2	-2.8	D-lactate dehydrogenase, mitochondrial
PHM6	-2.8	Protein predicted to have a role in phosphate metabolism
YOR225W	-1.9	Protein of unknown function
YER053C-A[a]	-2.7	Protein possibly involved in tolerance of high salt, osmotic stress, and low temperature
ERR1	-2.5	Enolase-related subtelomeric sequence, identical to Err2p
YLR099W-A	-1.8	Protein of unknown function
YLR346C	-1.8	Protein of unknown function
GAS3	-2.9	Protein with similarity to Gas1p
YEL073C	-2.2	Protein of unknown function
CPR5	-2.8	Cyclophilin (peptidylprolyl cis-trans isomerase or PPIase) of the endoplasmic reticulum
YAR028W	-1.7	Protein with strong similarity to subtelomerically-encoded proteins
YAL061W	-1.7	Member of the zinc-containing alcohol dehydrogenase family
YBR187W	-1.7	Protein of unknown function, contains six potential transmembrane segments
YJL217W	-2.5	Protein of unknown function
YJL200C	-1.9	Protein with similarity to aconitase, has potential mitochondrial transit peptide
YGL117W	-2.2	Protein of unknown function
YHR140W	-2.0	Cytoplasmic protein of unknown function
FMP12	-1.8	Member of the gamma-butyrobetaine hydroxylase family
TIP41	-4.3	Negative regulator of TOR signaling pathway
MRPL44	-2.2	Mitochondrial ribosomal protein of the large subunit (YmR44)
YFR055W	-1.8	Protein with similarity to E. coli cystathionine beta-lyase
RIB4	-4.9	Riboflavin biosynthesis pathway enzyme
REC8	-2.4	Protein required for meiosis and sporulation
RNR2	-2.2	Component of ribonucleotide reductase (ribonucleoside-diphosphate reductase)
SPL2	-3.1	Putative inhibitor of Pho80p-Pho85p cyclin-dependent protein kinase
URA1	-2.4	Dihydroorotate dehydrogenase, fourth step in pyrimidine biosynthesis pathway
AAH1[a]	-1.7	Adenine deaminase (adenine aminohydrolase)
PDH1	-1.8	Protein possibly involved in propionate utilization
ADH5	-2.4	Alcohol dehydrogenase, zinc-dependent, also called formaldehyde dehydrogenase
IMD2	-2.7	Inosine-5'-monophosphate dehydrogenase

a) Core Environmental Stress Response Genes

b) Genes with orthologs in *S. pombe* that show similar expression changes when *S. pombe* is exposed to 2 μM $CuSO_4$
