# Supplementary material for: Global transcriptional responses of fission and budding yeast to changes in copper and iron levels: a comparative study
Source: Genome Biol. 2007 May 3;8(5):R73. doi: 10.1186/gb-2007-8-5-r73 (PMC1929147; doi:10.1186/gb-2007-8-5-r73)
Supplement: Additional data file 2 — Provided is a table summarizing the sequences of all qPCR primers used. [file gb-2007-8-5-r73-S2.pdf]

Supplementary table 5. Quantitative real-time PCR primers

**Gene**	**Forward primer (5' ➔ 3')**	**Reverse primer (5' ➔ 3')**
actin	GCAAGCGTGGTATTTTGACCTT	GCAACACGAAGCTCGTTGTAGA
ctr4	CGTAGATTTTCTCCCGCTAGCA	GCAGGACCGAAGAAAATGAAGA
ctr5	GATGAGTATGAGTGGCATGAGTGGC	CCAGGATTTAGCCAGGAAACACG
ctr6	AGAGGTTATGCTGGTCAGCAGTCTG	CGTTGTAGGTCATGGCAACCAAC
fio1	TTTTCTCCTTCCCCATTCTCGG	TCCATCAGGGTCGACGTCGTAA
fip1	TTCGACGGGAGAAGAAGACTCGC	TCCCGTGCAATCCTCCTCCTCT
frp1	GATCGTGGCTGCAGAATGCTA	TTTGGACGAGGCCCTTTCA
ish1	AGTTTCGACATAGTCGCCTGCTGA	GGATTTGGCACTCGCGACGTA
isu1	AGCGCTTTTAATGGCATCCTCG	TGCCTCGTCATCCTACGTTACCACT
pex7	GTCACCCATTATGGATTAGGCGGA	AGACCAGCGCACACCAAACAAA
ppr12	TGAGACATCACCTGCTCCAG	TCCGGTGACTAGGTGAAACC
rds12	CCAAGGCTTGCTCTTACCAG	GACACCGGATTCACTCCACT
sib1	ATCCATCATGCCATGAACAGGG	CGCAGTTTTTGCTCGCCTTCT
sib2	TGGATTTGGTCCCGCTAGTCTTTC	CATACCCTCATGCCAGGAAAACGT
sid4	AGATGCAGATCCGTCTCGTACCG	CAAGTTGATGACGCGGATCCAG
SPAC15E1.02c	ACGACGTACGCCGTAACCGATC	TGGATTGCCCGACGTAGTCGA
SPAC1F8.02c	GGAATCAGAAGAATCAGCCGATGC	GGCGGCTGCAATGAATTCAGAT
SPAC3G6.05	GTTTAGTCGTTTTGCAACCCGC	TGGGCGACAGCATCGGATAT
SPAC458.03	GTAACCCGTTAGCCTTTGACGTTC	GGCTCCAAGTCATCTCCCCTTATT
SPAC56E4.03	AGGTTGTAGCCCCTGGTTCT	GAACAACGGTTGAGCAGTGA
SPBC1271.07c	TTATGCTAGTTGCAAGGGACGAGC	TCAGACCTCGACTCTCTGGCCTAA
SPBC27B12.03c	ATCCTTCTTGCTGGCATAAGTGCG	GCTCATCACGCTGCTCACCATATG
SPBC887.17	ATTGCCTCAATGGGTACGTC	GGTGGCATTGTCCATAGCTT
SPBC947.05c	CGATTGCCCTGATTTTCGTGCT	CCAGTGTCTCAGCGCCAGATAAACT
SPBPB2B2.05	CAAGGCAGGTGGTTGTCCAATT	CCACGACATATTCCAAGGATAGGG
srx1	ATTGGCAGAAGCACCCAGATACAG	GGCTGTCATCGACTACGTGCTCA
str1	CAGAGGCGAGCAATATAATTGGGG	TGGGTGTAACGGTACTAACAAGGGC
str3	GGCTCCATCACGAATTTCTGTGC	GGCAATCGCGTCTCCAATTTTC
vps53	GCCTGTCAGAAAGGCTTCAAGAGTC	CGGCTACTGATTGCAGGGTTGA
